# Effectiveness, safety, and biomarker dynamics of lecanemab in Chinese Alzheimer's disease population: a multicenter real‐world study

**DOI:** 10.1002/alz.71231

**Published:** 2026-03-13

**Authors:** Wang Liao, Qun Yu, Bin Chen, Hualin Chen, Liangyu Zou, Haiqun Xie, Haobo Chen, Chenyang Wang, Yang Li, Xiaoya Gao, Hongbo Guo, Ying Yang, Ziyu She, Qinggan Zeng, Zengqiang Zhang, Guihua Li, Shujun Feng, Han Lin, Jipeng Ouyang, Junyan Liang, Wenling Ao, Qiong Zeng, Zhou Liu, Hanyu Weng, Hongqiang Huang, Feiqi Zhu, Runni Liu, Yingte Wang, Dapeng Yu, Xue Li, Yingren Mai, Shengnan Jiang, Yu Tu, Zhiming Li, Jun Liu, for the Alzheimer's Disease Neuroimaging Initiative

**Affiliations:** ^1^ Department of Neurology Institute of Neuroscience, the Second Affiliated Hospital of Guangzhou Medical University Guangzhou Guangdong China; ^2^ Department of Neurology Shenzhen People's Hospital (First Affiliated Hospital of Southern University of Science and Technology), Second Clinical College, Jinan University Shenzhen Guangdong China; ^3^ Department of Neurology Foshan Hospital of Sun Yat‐Sen University Foshan Guangdong China; ^4^ Department of Neurology Guangzhou First People's Hospital South China University of Technology Guangzhou Guangdong China; ^5^ Department of Geriatrics Zhujiang Hospital Southern Medical University Guangzhou Guangdong P.R. China; ^6^ Department of Neurology, Department of Pediatric Neurology Zhujiang Hospital of Southern Medical University Guangzhou Guangdong China; ^7^ Department of Neurosurgery Center The National Key Clinical Specialty Guangdong Provincial Key Laboratory on Brain Function Repair and Regeneration The Neurosurgery Institute of Guangdong Province Zhujiang Hospital Southern Medical University The Engineering Technology Research Center of Education Ministry of China on Diagnosis and Treatment of Cerebrovascular Disease Guangzhou China; ^8^ Department of Neurology the First Affiliated Hospital of Jinan University Medical College Guangzhou Guangdong China; ^9^ Department of Neurology Zhongshan People's Hospital Shanghai Guangdong China; ^10^ Department of Neurology Heyou Hospital Foshan City Guangdong China; ^11^ Hainan Hospital of Chinese PLA Genera Hospital Sanya China; ^12^ Department of Neurology The Affiliated Guangdong Second Provincial General Hospital of Jinan University Guangzhou Guangdong China; ^13^ Department of Neurology Guangdong Provincial People's Hospital Guangzhou Guangdong China; ^14^ Department of Neurology Houjie Hospital of Dongguan Dongguan Guangdong China; ^15^ Department of Neurology The Eighth Affiliated Hospital of Southern Medical University (The First People's Hospital of Shunde Foshan) Guangdong China; ^16^ The International Medical Department Shenzhen Hospital, Southern Medical University Shenzhen China; ^17^ Department of Neurology the First Affiliated Hospital of Shantou University Medical College Shantou China; ^18^ Department of Neurology The Affiliated Hospital of Guangdong Medical University Zhanjiang Guangdong China; ^19^ Department of Neurology Faculty of Neurology Dongguan People's Hospital (Affiliated Dongguan Hospital South Medical University) Guangdong Medical University Dongguan Guangdong China; ^20^ Department of Geriatrics The Second Clinical College of Guangzhou University of Chinese Medicine Guangzhou China; ^21^ Cognitive Impairment Ward of Neurology Department The Third Affiliated Hospital of Shenzhen University Medical College Shenzhen Guangdong China; ^22^ Department of Neurology Zhuhai Hospital of Integrated Traditional Chinese and Western Medicine Zhuhai Guangdong China; ^23^ Department of Radiology the Second Affiliated Hospital of Guangzhou Medical University Guangzhou Guangdong China; ^24^ Department of Nuclear the Second Affiliated Hospital of Guangzhou Medical University Guangzhou Guangdong China; ^25^ Department of Neurology Zhuhai People's Hospital Zhuhai Guangdong China

**Keywords:** Alzheimer's disease, amyloid‐related imaging abnormalities (ARIA), real‐world study, lecanemab, plasma biomarkers

## Abstract

**BACKGROUND:**

Lecanemab, an anti–amyloid beta (Aβ) protofibril antibody, was introduced in China in 2024, but its real‐world performance remains unknown.

**METHODS:**

In this prospective, multicenter study across 21 sites, 261 Alzheimer's disease patients (mild cognitive impairment to moderate dementia) received biweekly lecanemab (10 mg/kg). A matched Alzheimer's Disease Neuroimaging Initiative (ADNI) cohort served as comparator. Cognitive tests, plasma biomarkers, and optional amyloid/tau positron emission tomography (PET) were assessed over 6 months.

**RESULTS:**

Lecanemab significantly attenuated cognitive decline versus ADNI. Plasma Aβ42, Aβ40, phosphorylated tau 217 (p‑tau217), glial fibrillary acidic protein (GFAP), and ratios showed robust changes; a p‑tau217 reduction correlated with amyloid PET clearance (mean −22.1 Centiloid; 29.2% turned amyloid‐negative). Apolipoprotein E *(* *APOE)* ε4 non‐carriers showed greater improvements. Infusion reactions occurred in 11.1% and amyloid‐related imaging abnormalities in 9.2% (1.6% symptomatic), with no stage‐related safety differences.

**CONCLUSION:**

Lecanemab was effective and well tolerated in real‐world Chinese patients. Plasma p‑tau217 may serve as a sensitive, minimally invasive treatment‐response biomarker.

## Highlights

- This is the first large‐scale real‐world evaluation of lecanemab in Chinese Alzheimer's disease patients, demonstrating slowed cognitive decline and robust biomarker improvements.
- Plasma p‐tau217 reduction strongly correlated with amyloid PET clearance, supporting its use as a treatment response marker.
- Lecanemab was well tolerated across all AD stages, with no increased adverse events in moderate dementia patients.

## 1 INTRODUCTION

Alzheimer's disease (AD) is a progressive neurodegenerative disorder characterized by cognitive impairment, memory loss, and gradual decline in daily functioning. The accumulation of amyloid beta (Aβ) is recognized as a pivotal pathological driver of disease onset and progression.^1^ Lecanemab, a humanized monoclonal antibody that selectively targets soluble Aβ protofibrils, has emerged as a promising disease‐modifying therapy for early‐stage AD. By facilitating the clearance of soluble amyloid aggregates, lecanemab may reduce neurotoxicity and slow disease progression.^2^

The clinical efficacy and safety of lecanemab have been rigorously validated in phase 2b (BAN2401‐G000‐201) and phase 3 Clarity AD trials,^3^ which demonstrated significant reductions in amyloid burden alongside a slowed rate of cognitive decline. These benefits were most pronounced in patients with mild cognitive impairment (MCI) or mild AD, with a modest yet statistically significant deceleration in disease progression. Since its availability in China in June 2024, lecanemab has entered routine clinical use; however, robust real‐world evidence regarding its safety and effectiveness in Chinese and broader Asian populations remains limited.

A distinctive feature of lecanemab's pharmacological profile is its preferential binding to protofibrils over amyloid plaques, potentially enhancing clearance of neurotoxic Aβ species while mitigating plaque‐related adverse effects.^4^ In parallel, biomarkers such as phosphorylated tau (p‐tau), neurofilament light chain (NfL), and glial fibrillary acidic protein (GFAP) have gained recognition as objective indicators for evaluating disease‐modifying effects.^5^ Emerging evidence suggests that tau reduction often accompanies amyloid clearance, consistent with the interrelationship between Aβ and tau pathologies in AD progression.^6^, ^7^

Despite encouraging preliminary results, questions remain regarding the long‐term durability of lecanemab's benefits. While amyloid reduction correlates with slower cognitive decline, its impact on quality of life is yet to be fully established. In the United States, the US Food and Drug Administration (FDA) has approved the once every four weeks maintenance dosing regimen for patients who complete the standard 18‐month course of lecanemab; however, specific guidelines for those achieving amyloid positron emission tomography (PET) negativity within 6 months of therapy are currently unavailable. Early transition to a maintenance regimen could substantially reduce treatment costs – an especially important consideration in resource‐limited settings such as China.

Furthermore, recent international consensus statements have incorporated plasma biomarker testing into the AD diagnostic framework,^8^ offering an accessible and minimally invasive monitoring option. Given that lecanemab administration requires biweekly infusions over 18 months, adherence can be challenging. The use of ultrasensitive detection techniques such as single‐molecule array (Simoa) for plasma biomarkers could facilitate reliable, real‐time monitoring, improving patient confidence and compliance.^9^

**RESEARCH IN CONTEXT**

1. **Systematic review**: We systematically searched PubMed, Embase, and ClinicalTrials.gov from database inception to September 14, 2025, using the terms “lecanemab,” “amyloid‐beta,” “Alzheimer's disease,” “real‐world,” and “China,” with no language restrictions. Pivotal phase 3 trials, including CLARITY AD, demonstrated that lecanemab reduces amyloid burden and slows cognitive decline in early AD. However, these trials were conducted predominantly in non‐Chinese populations under controlled conditions and did not assess large‐scale real‐world effectiveness, detailed biomarker dynamics, or optimal monitoring strategies – particularly the role of plasma p‐tau217 in treatment response evaluation.
2. **Interpretation**: Our prospective, multicenter, large‐scale, real‐world study is the first to evaluate lecanemab in Chinese patients across MCI to moderate dementia. We observed significantly attenuated 6‐month cognitive decline compared with a matched ADNI cohort, robust plasma biomarker changes – especially p‐tau217 reduction correlated with amyloid PET clearance – and favorable safety across disease stages. These findings extend lecanemab's evidence base from controlled trials to routine clinical practice in China and support plasma p‐tau217 as a sensitive, minimally invasive treatment response marker.
3. **Future directions**: Future studies should validate the predictive value of plasma p‐tau217 and other blood biomarkers for long‐term clinical outcomes, determine optimal monitoring intervals, and explore cost‐effectiveness in diverse healthcare settings. Randomized controlled trials in Chinese and other underrepresented populations, including those at more advanced disease stages, are needed to confirm the generalizability of these findings. Moreover, investigations into combination therapies and individualized treatment algorithms based on biomarker profiles could further optimize anti‐amyloid interventions.

## 2 METHODS

### 2.1 Subject

**FIGURE 1:**
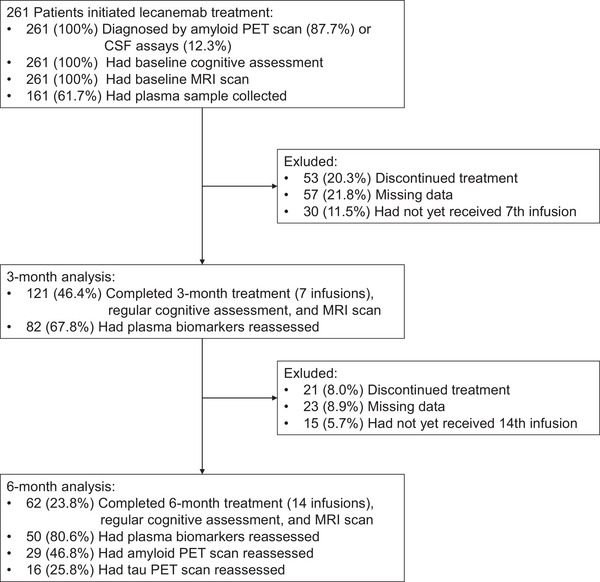
Flowchart of patient screening and enrollment in study.

This is a 6‐month, multicenter, prospective, real‐world study to evaluate the effectiveness and safety of lecanemab in AD. Patients in this study received lecanemab treatment (one infusion every 2 weeks). Aβ was confirmed through PET or cerebrospinal fluid (CSF) testing (Figure 1). Patients with magnetic resonance imaging (MRI) contraindications (e.g., claustrophobia or metal implants) or those without supporting Aβ PET or CSF Aβ‐positive results were excluded. Patients with severe hearing or vision impairment, which would prevent them from cooperating with assessments, were also excluded. Demographic information, including gender, age, and years of education, was collected at baseline for all patients. Additionally, all adverse events occurring between lecanemab infusions were recorded from one infusion to the next. A total of 261 patients were enrolled in the study. We also selected 76 patients from the Alzheimer's Disease Neuroimaging Initiative (ADNI) as an external control group for comparison of longitudinal cognitive scale data. Within the matched ADNI cohort of 76 individuals, 46 had available Aβ PET results, of which 41 were positive. These participants underwent at least 2 years of follow‐up, ensuring robust diagnoses. The ADNI was launched in 2003 as a public–private partnership, led by Principal Investigator Michael W. Weiner, MD. The goal of ADNI include validating biomarkers for clinical trials, improving the generalizability of ADNI data by increasing diversity in the participant cohort, and to provide data concerning the diagnosis and progression of AD to the scientific community.^10^

### 2.2 PET imaging

PET images were scanned with the GE Discovery 710 PET/CT scanner at each center. 18F‐Florbetapir (FBP) and/or 18F‐flortaucipir (FTP) scans were acquired before first infusion to verify the deposition of AD pathology and acquired for consent participants who finished 6 months of treatment. The tracers were provided by Guangdong Cyclotron Medicine Technology Co., Ltd. All PET images were visually analyzed independently by two senior nuclear medicine physicians, who classified the amyloid or tau deposition as negative or positive, based on a published staging standard.^11^, ^12^ Any disagreement between the two PET physicians was proceeded to consult a third senior assessor. The composite visual analysis was used to diagnose AD according to the 2018 National Institute on Aging–Alzheimer's Association framework and the 2023 Alzheimer's Association Workgroup revised criteria,^1^, ^13^ requiring the presence of Aβ and/or tau deposition. PET images were then coregistered to participants’ recent 1‐month MRI T1‐weighted images and matched the Montreal Neurological Institute (MNI) template, with standardized uptake value (SUV) extracted from normalized cortex and whole cerebellum (FBP) or cerebellar cortex (FTP) by the Hetu Neuroimaging Processing Software (Shenzhen Hetu Medical Technology Co.). Subsequently the Aβ PET SUV ratios (SUVRs) were converted to Centiloid (CL) under the verified data processing flow, standard volume of interest (VOI) masks, and quality control check as the Centiloid Project.^14^, ^15^, ^16^, ^17^ The pathology‐based standard 24.1 CL was used as the Aβ clearance threshold at 6 months.^16^, ^18^, ^19^

### 2.3 MRI scanning

MRI scans were conducted at baseline and prior to the first, fifth, seventh, and 14th doses of lecanemab infusion. The imaging protocol included T1‐weighted 3D, T2 Fluid‐Attenuated Inversion Recovery (T2 FLAIR), and Susceptibility Weighted Imaging (SWI). T1 3D imaging provided high‐resolution structural images of the brain, enabling the assessment of anatomical changes such as brain atrophy. T2 FLAIR was used to detect white matter lesions, edema, and other pathological changes, while SWI helped identify microbleeds and small vascular abnormalities.

MRI scans before the fifth, seventh, and 14th doses of lecanemab were performed to monitor any potential treatment‐related structural changes or adverse effects. Each MRI scan review is independently conducted by two senior radiologists to ensure the timely detection of amyloid‐related imaging abnormalities (ARIA) and prompt intervention. These evaluations are essential for tracking the progression of neurodegenerative pathology and ensuring that any structural changes or abnormalities in the brain are thoroughly documented and linked to the treatment protocol.

### 2.4 Plasma biomarkers

Six plasma proteins were measured using single‐molecule array (Simoa) technology provide by Fuzhou Ribose Medical Laboratory (Fuzhou, China), including Aβ42, Aβ40, p‐tau217, p‐tau181, NfL, and GFAP. Aβ42, Aβ40, GFAP, and NfL were measured using the Neurology 4‐Plex E Assay Kit (Simoa, Quanterix, 103670), while p‐tau217 was detected with the ALZPath p‐tau 217 Advantage PLUS kit (Simoa, Quanterix, 104570), and p‐tau181 was measured using the p‐tau181 Assay Kit V2.1 (Simoa, Quanterix, 104111). The assessments were conducted before treatment, and at 3 and 6 months after the initiation of therapy.

### 2.5 Neuropsychological assessments

Cognitive function was assessed at baseline using several standard scales: the Mini‐Mental State Examination (MMSE), the Alzheimer's Disease Assessment Scale (ADAS)‐Cognitive subscale 14, the Clinical Dementia Rating (CDR) scale, the ADAS MCI Activities of Daily Living Scale (ADAS‐MCI‐ADL) scale, the Hamilton Depression Rating Scale (HAMD), and the Neuropsychiatric Inventory.^20^ These assessments provided a comprehensive evaluation of cognitive function, daily living abilities, depressive symptoms, and behavioral disturbances, which are crucial for understanding a patient's condition at the start of the study. Follow‐up assessments were conducted at 3 and 6 months after baseline to monitor changes in cognitive performance and disease progression. Every cognitive assessment was conducted by two professionals who had received training in cognitive assessment. The two individuals took turns to act as assessors and quality controllers to ensure the reliability of each assessment.

### 2.6 Statistical analysis

Between‐group comparisons for continuous variables were made using independent sample *t*‐tests (for two groups) or one‐way ANOVA (for three or more groups). The Kruskal–Wallis test was used for non‐normally distributed data. Categorical variables were compared using chi‐squared tests or Fisher's exact tests, as appropriate. Post hoc analyses were performed using Tukey's Honestly Significant Difference test for normally distributed data or Dunn's test with Bonferroni adjustment for non‐parametric data. Longitudinal changes in cognitive scores and plasma biomarkers were analyzed using paired *t*‐tests or Wilcoxon signed‐rank tests for within‐group comparisons at 3 and 6 months. Propensity score matching (PSM) was employed to match the lecanemab cohort with the ADNI control group based on age, gender, education, and apolipoprotein E *(* *APOE)* ε4 carrier status. A 2:1 nearest‐neighbor matching algorithm with a caliper of 0.2 was used to ensure comparability. Correlation analyses between changes in Aβ PET, plasma biomarkers, and cognitive scores were performed using Spearman correlation coefficients. A *p* value < 0.05 was considered statistically significant. All statistical analyses were performed using PyCharm 2024.3.4 and R version 4.5.1.

## 3 RESULTS

### 3.1 Demographic and clinical parameters

The study enrolled 261 participants diagnosed with AD by PET (87.7%) or CSF (12.3%), primarily elderly (73.6% aged over 65 years old) with a female predominance (59.0%) (Figure 1). Nearly half of patients carried at least one *APOE* ε4 allele (47.9%), while cardiovascular risk factors were common (24.9% hypertension). Standard dementia treatments were widely used, with acetylcholinesterase inhibitors (48.3%) and memantine (28.4%) being the most frequently prescribed medications, though 37.2% received no documented concomitant therapy (Table 1). The mean baseline MMSE score was 18.22, and 82.4% participants had MCI or a mild dementia stage of disease, while 17.6% received a baseline CDR‐global score (GS) of 2 (Table 1).

**TABLE 1:** Characteristics of participants at baseline.

Characteristic	Participants (*N* = 261)
**Age, mean (SD)**	69.74 (8.92)
**Education, *N* (%)**	
0 to 12 years	195 (74.7)
>12 years	66 (25.3)
**Gender, *N* (%)**	
Female	154 (59.0)
Male	107 (41.0)
**Body mass index, mean (SD)**	22.40 (2.99)
***APOE* ε4 status, *N* (%)**	
Non‐carrier	105 (40.2)
Carrier	125 (47.89)
Heterozygotes	105 (40.2)
Homozygotes	20 (7.7)
**Current use of medication, *N* (%)**	
Acetylcholinesterase inhibitor	126 (48.3)
NMDA receptor antagonist	74 (28.4)
Antiplatelet agents	30 (11.5)
Anticoagulant	2 (0.7)
Antihypertensives	65 (24.9)
Antidiabetic agents	30 (11.5)
Antipsychotics	20 (7.7)
None	97 (37.2)
**Comorbidities, *N* (%)**	
Hypertension	65 (24.9)
Diabetes	30 (11.5)
CVDs	27 (10.3)
Anxiety	5 (1.9)
Depression	3 (1.1)
Atrial fibrillation	2 (0.7)
None	137 (52.5)
**Clinical outcome**	
MMSE score, mean (SD)	18.22 (6.10)
CDR‐GS, *N* (%) 0.5, MCI	107 (41.0)
1, mild AD	108 (41.4)
2, moderate AD	46 (17.6)
**Microhemorrhage, *N* (%)**	37 (14.2)
**Superficial siderosis, *N* (%)**	4 (1.5)

Abbreviations: AD, Alzheimer's disease; *APOE*, apolipoprotein E; CDR‐GS, Clinical Dementia Rating Scale‐Global Score; CVD = cardiovascular disease; MMSE, Mini‐Mental State Examination; NMDA, N‐methyl‐D‐aspartate.

### 3.2 Cognitive function assessment

**FIGURE 2:**
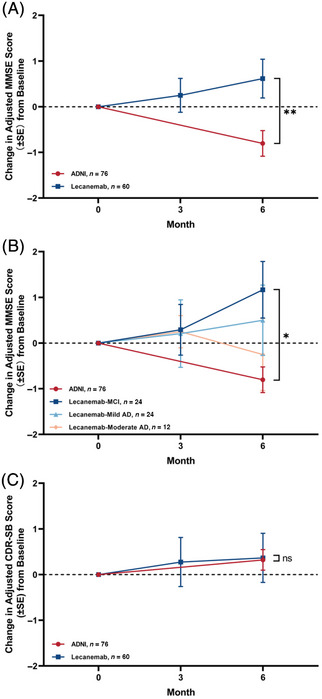
Cognitive changes over 6 months in lecanemab and Alzheimer's Disease Neuroimaging Initiative (ADNI) cohorts. (A) Changes in adjusted Mini‐Mental State Examination (MMSE) scores from baseline in both cohorts. (B) Changes in adjusted MMSE scores for the lecanemab cohort by disease severity and the ADNI cohort. (C) Changes in adjusted Clinical Dementia Rating Sum of Boxes (CDR‐SB) scores from baseline in both cohorts. The lecanemab and ADNI cohorts were matched via propensity score matching on age, gender, education, and *APOE* ε4 status. In the lecanemab cohort, patients were stratified into mild cognitive impairment (MCI), mild Alzheimer's disease (AD), and moderate AD groups based on CDR‐global score (GS) scores of 0.5, 1, and 2, respectively. *p < 0.05, **p < 0.01.

Cognitive function was evaluated at 3 and 6 months of treatment using two primary clinical scales: the MMSE and the CDR Sum of Boxes (CDR‐SB). PSM was applied to select comparable participants from the ADNI cohort, resulting in matched samples of 76 patients from ADNI and 60 from the lecanemab cohort. After 6 months of lecanemab treatment, patients demonstrated a significant improvement in MMSE scores compared with the matched ADNI cohort, with mean changes ±SE of 1.45 ±0.51 (*p* < 0.01), respectively. When participants were stratified by baseline disease severity (MCI, mild AD, and moderate AD), a significant between‐group difference from the ADNI cohort was observed only in the MCI subgroup at 6 months (*p* = 0.02), while no significant differences were detected between the ADNI cohort and either the mild or moderate AD subgroups. When using CDR‐SB as the evaluation metric, lecanemab‐treated patients showed a pronounced improvement relative to the matched ADNI cohort, whereas no significant differences were observed across other severity subgroups. (Table 2, Figure 2, Table S1).

### 3.3 Changes in plasma biomarkers after 6 months of lecanemab treatment

In the cohort of 82 patients assessed at baseline and after 3 months of treatment, plasma Aβ42 and Aβ40 levels increased significantly compared with baseline (Aβ42: 4.794 ± 2.257 pg/mL to 6.004 ± 1.964 pg/mL, 95% CI: 0.735 to 2.048, *p* < 0.01; Aβ40: 95.106 ± 42.574 pg/mL to 128.427 ± 45.697 pg/mL, 95% CI: 20.053 to 46.960, *p* < 0.01), while the Aβ42/40 ratio remained unchanged (*p* = 0.86). Among the single biomarkers, a significant decrease was observed in NfL (27.307 ± 11.614 pg/mL to 25.057 ± 10.407 pg/mL, 95% CI: −5.063 to 0.338, *p* = 0.03). In contrast, no statistically significant changes were found in p‐tau217 (*p* = 0.05), p‐tau181 (*p* = 0.34), or GFAP (*p* = 0.06). All composite indices showed significant decreases, including p‐tau181/Aβ42 (0.672 ± 0.411 to 0.476 ± 0.249, 95% CI: −0.302 to −0.115, *p* < 0.01), p‐tau217/Aβ42 (0.237 ± 0.163 to 0.159 ± 0.103, 95% CI: −0.123 to −0.046, *p* < 0.01), GFAP/Aβ42 (57.744 ± 32.535 to 41.784 ± 24.692, 95% CI: −26.870 to −9.693, *p* < 0.01), and NfL/Aβ42 (5.756 ± 2.977 to 4.690 ± 2.850, 95% CI: −1.885 to 0.257, *p* < 0.01).

Among the 50 patients who completed 6 months of treatment, comparisons between baseline and the 6‐month time point revealed significant increases in plasma Aβ42 (from 4.562 ± 2.113 pg/mL to 5.752 ± 2.464 pg/mL, 95% CI: 0.416 to 1.541, *p* < 0.01) and Aβ40 (from 88.300 ± 42.114 pg/mL to 122.395 ± 51.634 pg/mL, 95% CI: 16.771 to 45.474, *p* < 0.01). The Aβ42/40 ratio did not change significantly (*p* = 0.34). Significant reductions were observed in p‐tau217 (from 1.183 ± 0.730 pg/mL to 0.912 ± 0.595 pg/mL, 95% CI: −0.436 to −0.186, *p* < 0.01) and GFAP (from 259.096 ± 112.570 pg/mL to 215.714 ± 122.685 pg/mL, 95% CI: −71.226 to −15.200, *p* < 0.01). Changes in p‐tau181 (*p* = 0.32) and NfL (*p* = 0.17) were not statistically significant. All composite indices showed significant decreases: p‐tau181/Aβ42 (from 0.696 ± 0.369 to 0.463 ± 0.218, 95% CI: −0.251 to −0.010, *p* < 0.01), p‐tau217/Aβ42 (from 0.255 ± 0.153 to 0.158 ± 0.082, 95% CI: −0.111 to −0.006, *p* < 0.01), and GFAP/Aβ42 (from 61.277 ± 35.670 to 43.543 ± 34.869, 95% CI: −26.487 to −8.027, *p* < 0.01), and NfL/Aβ42 (5.770 ± 3.021 to 4.838 ± 3.538, 95% CI: −2.137 to 0.242, *p* < 0.01).

**FIGURE 3:**
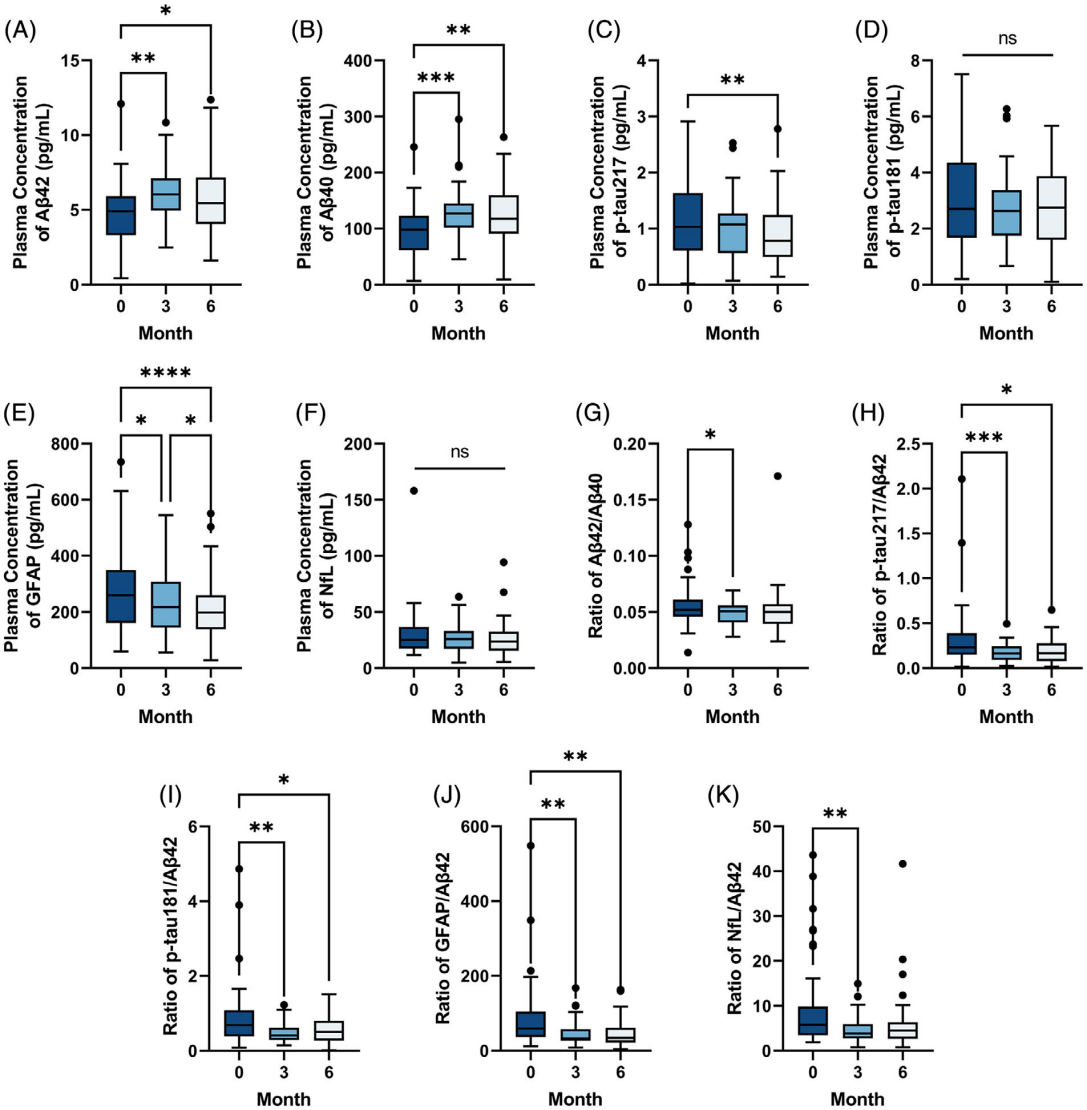
Temporal changes in plasma biomarkers during lecanemab treatment. (A–K) Box plots show distribution of single biomarkers (Aβ42, Aβ40, p‐tau217, p‐tau181, GFAP, NfL) and composite ratios (Aβ42/40, p‐tau217/Aβ42, p‐tau181/Aβ42, GFAP/Aβ42, NfL/Aβ42) measured at baseline, 3 months, and 6 months (*n* = 50). Data are presented as median (center line), IQR (box limits), and 1.5 × IQR whiskers. Statistical significance was assessed by a linear mixed‐effects model with Tukey's Honestly Significant Difference test. Significant comparisons from the post hoc test are indicated on the figure by asterisks (*p < 0.05, **p < 0.01). Aβ, amyloid beta; GFAP, glial fibrillary acidic protein; IQR, interquartile range; NfL, neurofilament light chain; p‐tau, phosphorylated tau.

Collectively, across both time points, Aβ42, Aβ40, p‐tau217, and GFAP were single biomarkers with consistent and significant post‐treatment improvement, whereas composite indices incorporating Aβ42 in the denominator captured additional significant changes undetected in single biomarker analyses, suggesting that such composite measures may offer greater sensitivity in detecting treatment‐related effects and that plasma biomarker profiling could serve as a valuable tool for monitoring therapeutic responses in patients receiving lecanemab (Table 2, Figure 3, Table S2).

**TABLE 2:** Outcomes after 3 and 6 months of lecanemab treatment.

Outcomes	Before treatment	After 3 months of treatment	*P*	Before treatment	After 3 months of treatment	After 6 months of treatment	*P*
	(*N* = 82)			(*N* = 50)			
**Clinical outcome, mean (SD)**							
CDR‐SB	5.44 (4.10)	5.76 (4.29)	0.24	5.36 (4.01)	5.63 (4.19)	6.02 (4.29)	0.04
MMSE score	17.44 (6.62)	17.02 (6.57)	0.40	17.08 (6.46)	17.08 (6.50)	17.58 (7.29)	0.41
**Plasma biomarkers, mean (SD)**							
Aβ42 (pg/mL)	4.794 (2.257)	6.004 (1.964)	<0.01	4.562 (2.113)	5.920 (1.539)	5.752 (2.464)	<0.01
Aβ40 (pg/mL)	95.106 (42.574)	128.427 (45.697)	<0.01	88.300 (42.114)	125.063 (36.203)	122.395 (51.634)	<0.01
Aβ42/40	0.048 (0.010)	0.048 (0.010)	0.86	0.051 (0.010)	0.048 (0.009)	0.048 (0.012)	0.34
P‐tau217 (pg/mL)	1.080 (0.697)	0.958 (0.617)	0.05	1.183 (0.730)	0.974 (0.498)	0.912 (0.595)	<0.01
P‐tau181 (pg/mL)	2.889 (1.562)	2.774 (1.306)	0.34	3.038 (1.561)	2.834 (1.213)	2.793 (1.471)	0.32
GFAP (pg/mL)	253.374 (117.659)	232.700 (122.057)	0.06	259.096 (112.570)	239.362 (123.616)	215.714 (122.685)	<0.01
NfL (pg/mL)	27.307 (11.614)	25.057 (10.407)	0.03	27.885 (12.011)	25.425 (10.865)	23.668 (10.553)	0.17
P‐tau181/Aβ42	0.672 (0.411)	0.476 (0.249)	<0.01	0.696 (0.369)	0.463 (0.218)	0.595 (0.400)	<0.01
P‐tau217/Aβ42	0.237 (0.163)	0.159 (0.103)	<0.01	0.255 (0.153)	0.158 (0.082)	0.199 (0.177)	<0.01
GFAP/Aβ42	57.744 (32.535)	41.784 (24.692)	<0.01	61.277 (35.670)	40.587 (23.957)	43.543 (34.869)	<0.01
NfL/Aβ42	5.756 (2.977)	4.690 (2.850)	<0.01	5.770 (3.021)	4.983 (3.394)	4.838 (3.538)	<0.01

Abbreviations: Aβ, amyloid beta; CDR‐SB, Clinical Dementia Rating Scale‐Sum of Boxes; GFAP, glial fibrillary acidic protein; MMSE, Mini‐Mental State Examination; NfL, neurofilament light chain; p‐tau, phosphorylated tau; SD, standard deviation.

### 3.4 Changes in Aβ and tau PET at baseline and after 6 months of lecanemab treatment

**FIGURE 4:**
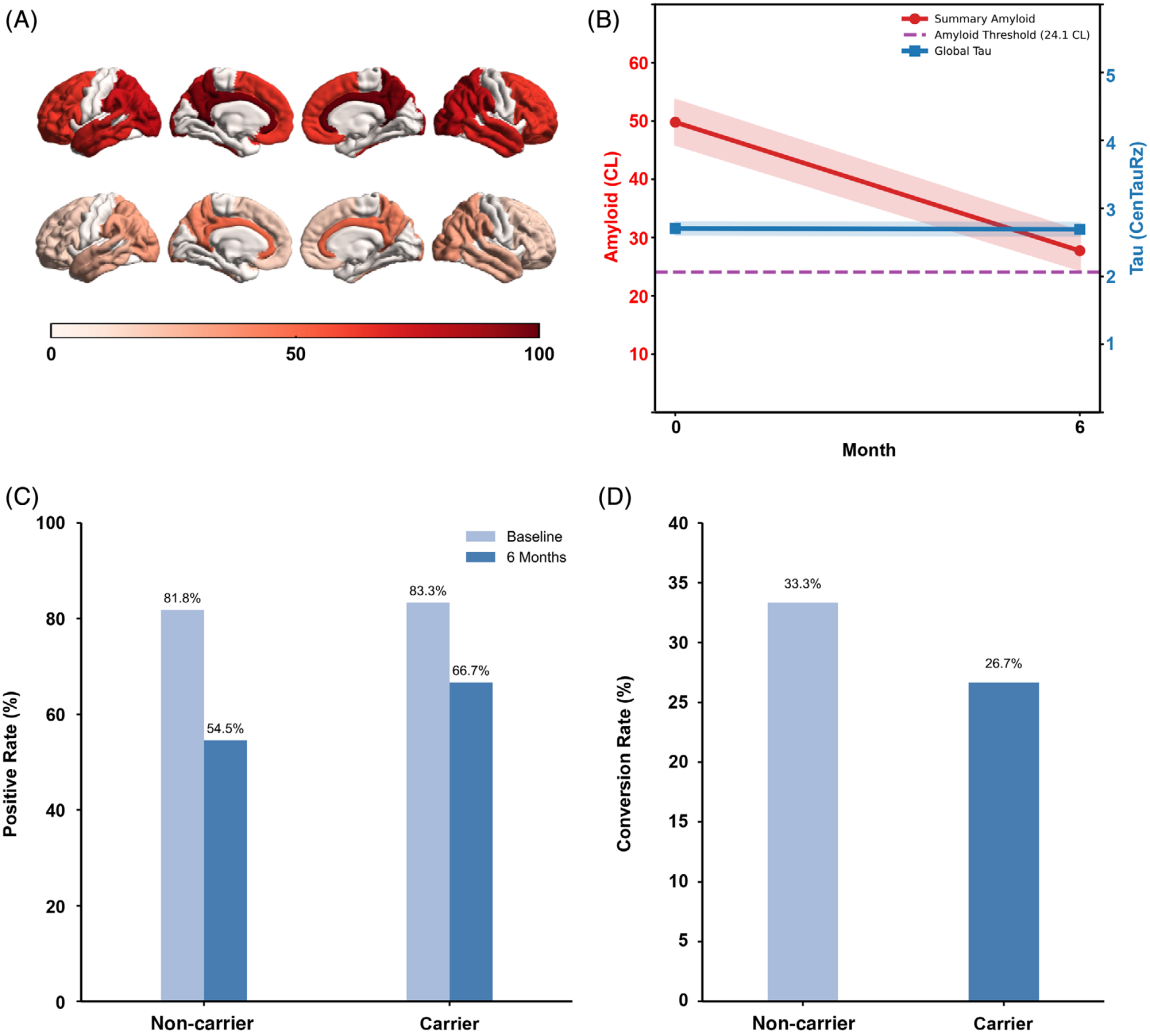
Changes in Aβ (*N* = 29) and tau (*N* = 16) PET at baseline and after 6 months of lecanemab treatment. (A) Brain surface maps depicting changes of Aβ burden in cortex between baseline and 6 months. (B) Quantitative PET measures of cortex Aβ (CL) and tau (SUVR) showing changes after 6 months. (C) Aβ PET CL value positivity in *APOE* ε4 carriers and non‐carriers, before and after treatment using 24.1 CL as threshold. (D) Comparison of proportion of patients converting from Aβ CL value positive to negative status between *APOE* ε4 carriers and non‐carriers. Aβ, amyloid beta; *APOE*, apolipoprotein E; CL, Centiloid; PET, positron emission tomography; SUVR, standardized uptake value ratio.

In the subgroup of 29 patients who underwent Aβ and 16 patients who had tau PET scans at baseline and after 6 months of lecanemab treatment, visual inspection of the brain surface maps (Figure 4A) revealed a marked reduction in Aβ load across multiple cortical regions following treatment. Quantitative PET analysis (Figure 4B) demonstrated a significant decrease in summary Aβ burden, with CL values declining from a mean of 49.79 ± 25.76 at baseline to 27.75 ± 22.12 after 6 months (difference: −16.51, 95% CI: −29.52 to −3.49, *p *< 0.05), approaching the conventional amyloid clearance threshold of 24.1 CL. In contrast, global tau burden showed minimal change over the same period, from baseline SUVR 1.35 ± 0.30 to 6 months 1.35 ± 0.32 (difference: −0.01, 95% CI: −0.07 to 0.06, *p* > 0.05). When stratified by *APOE* ε4 status, the conversion rate from amyloid CL value positive to negative status was higher in non‐carriers than in carriers, although the differences did not reach statistical significance (Figure 4C,D). These imaging findings indicate that lecanemab treatment was associated with a robust and regionally consistent reduction in cerebral Aβ burden over 6 months, without a corresponding short‐term change in global tau levels.^21^

### 3.5 Correlations between changes in Aβ PET, cognitive assessments, and blood biomarkers

**FIGURE 5:**
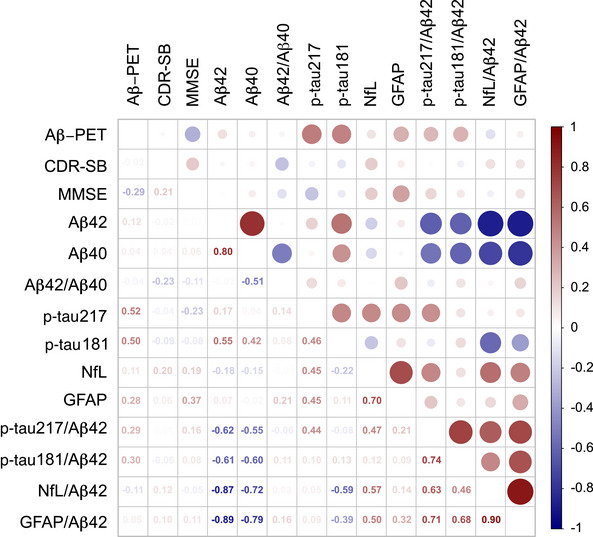
Correlations between changes in Aβ PET, cognitive assessments, and plasma biomarkers. Aβ, amyloid beta; CDR‐SB, Clinical Dementia Rating Scale‐Sum of Boxes; GFAP, glial fibrillary acidic protein; MMSE, Mini‐Mental State Examination; NfL, neurofilament light chain; p‐tau, phosphorylated tau.

In the 29 patients who completed 6 months of lecanemab treatment, longitudinal correlations were assessed between changes in amyloid burden, cognitive measures, and plasma biomarkers (Figure 5, Table S3). Changes in Aβ PET showed significant positive correlations with changes in both p‐tau217 (*r* = 0.52, *p* = 0.02) and p‐tau181 (*r* = 0.50, *p* = 0.02).^22^

Changes in cognitive measures showed limited correlations with biomarker changes in this short‐term period. MMSE changes showed a negative correlation with Aβ PET accumulation (*r* = −0.29, *p* = 0.16) and a positive correlation with GFAP increase (*r* = 0.28, *p* = 0.18), suggesting potential relationships that may become more pronounced with longer observation periods.

Plasma biomarkers demonstrated robust intercorrelations that may reflect treatment effects. The ratio‐based biomarkers p‐tau181/Aβ42 and p‐tau217/Aβ42 were strongly correlated with each other (*r* = 0.74, *p* < 0.01) and with measures of neuroaxonal injury and astroglial activation, including significant correlations with NfL/Aβ42 (*r* = 0.71, *p* < 0.01) and GFAP/Aβ42 (*r* = 0.63, *p* < 0.01).^23^ Additionally, NfL and GFAP showed a strong positive correlation (*r* = 0.70, *p* < 0.01), indicating concurrent reduction in neuroaxonal injury and astroglial activation during treatment.^24^

Taking into account the longitudinal trajectories of plasma biomarkers and their association with Aβ PET findings, p‐tau217 appears to be a more reliable marker for assessing response to lecanemab therapy.

### 3.6 Adverse events and ARIA

Across the entire cohort of patients who received lecanemab treatment, the most common adverse event was infusion‐related reaction (11.1%), followed by tremor (1.91%), dizziness (0.8%), and increased blood pressure or headache (both 0.4%).^25^ Less frequent events included subacute lacunar infarction (1.2%) and cerebral hemorrhage (0.8%). Serious adverse events were reported in four patients (1.6%), and in a small number of cases, these led to treatment discontinuation (Table 3).

**TABLE 3:** Adverse events (AEs) of participants after at least one infusion.

Event	After treatment
**AE, *N* (%)**	
Infusion‐related reaction	29 (11.1)
Tremor	5 (1.9)
Dizziness	2 (0.8)
Increased blood pressure	1 (0.4)
Headache	1 (0.4)
Subacute lacunar infarction	3 (1.1)
Cerebral hemorrhage	2 (0.8)
**Serious AE, *N* (%)**	4 (1.6)
**AE that led to discontinuation of treatment, *N* (%)**	5 (1.9)
**ARIA event (E or H** **), *N* (%)**	
**Total**	24 (9.2)
ARIA‐E	4 (1.6)
ARIA‐H	21 (8.0)
**Severity**	
Mild	18 (6.9)
Moderate	5 (1.9)
Severe	1 (0.4)
**Symptom status**	
Asymptomatic	20 (7.7)
Symptomatic	4 (1.6)
**Brain region**	
Frontal lobe	3 (1.1)
Parietal lobe	2 (0.8)
Temporal lobe	3 (1.1)
Occipital lobe	1 (0.4)
Cerebellum	5 (1.9)
Pon	1 (0.4)
Basal ganglia	2 (0.8)
Corona radiata	1 (0.4)
**CDR‐GS**	
0.5, MCI	7 (2.7)
1, mild AD	13 (5.0)
2, moderate AD	4 (1.6)
***APOE* ε4 status**	
Non‐carrier	14 (5.4)
Carrier	10 (3.8)
Heterozygous	6 (2.3)
Homozygous	4 (1.6)

Abbreviations: AD, Alzheimer's disease; *APOE*, apolipoprotein E; ARIA, amyloid‐related imaging abnormalities; ARIA‐E, amyloid‐related imaging abnormalities of edema/effusions; ARIA‐H, amyloid‐related imaging abnormality of microhemorrhages and hemosiderin deposits; CDR‐GS, Clinical Dementia Rating Scale‐Global Score; MCI, mild cognitive impairment.

ARIA occurred in 24 (9.2%) patients. Amyloid‐related imaging abnormalities of edema/effusions (ARIA‐E) was reported in four (1.6%) patients, and amyloid‐related imaging abnormality of microhemorrhages and hemosiderin deposits (ARIA‐H) was observed in 8.0% (mild 6.9%, moderate 1.9%, severe 1.4%), with 7.7% symptomatic and 1.6% symptomatic. Regarding the location of ARIA occurrence, we observed that it appears most frequently in the cerebellum. The distribution of ARIA events showed no significant differences across *APOE* ε4 status genotypes (non‐carrier, heterozygous, or homozygous). Importantly, in the subgroup with moderate AD, the incidence and severity of ARIA were comparable to earlier studies in early AD populations, indicating that lecanemab use in moderate AD does not increase the risk of ARIA.

## 4 DISCUSSION

In this prospective, multicenter real‐world study from China, lecanemab demonstrated substantial Aβ clearance and stabilization of cognitive decline over 6 months, with the most pronounced benefits observed in *APOE* ε4 non‐carriers.^26^ Because our trial was single‐arm, we matched untreated patients from the ADNI cohort and found that treated patients showed significantly slower cognitive decline compared with the natural cohort. However, when stratified by disease severity, patients with mild AD (CDR = 1) and moderate AD (CDR = 2) did not show significant differences at 6 months. This finding is consistent with Clarity AD, where treatment effects were most evident in the early symptomatic phase (MCI and very mild dementia), with attenuated benefit in more advanced stages. This may reflect the limited follow‐up period in our study and the possibility that structural neuronal damage in later stages reduces responsiveness to amyloid clearance.^27^

Among the 29 patients who underwent paired Aβ PET scans, 29.2% achieved significant amyloid clearance and converted to PET‐negative status, with amyloid burden falling below the clearance threshold of 24.1 CL. Notably, the proportion was 55% in Clarity AD at 18 months.^2^, ^3^ As sample size increases, this proportion may approach values reported in larger trials.

Plasma biomarker analysis revealed consistent increases in Aβ42 and Aβ40. Specifically, changes in Aβ PET burden were significantly correlated with changes in plasma p‐tau217 and p‐tau181, indicating a concurrent reduction in amyloid accumulation and tau phosphorylation following treatment.^28^ This coupling effect supports the hypothesis that lecanemab‐induced clearance of Aβ protofibrils may attenuate downstream tau pathology, consistent with the amyloid cascade framework.^22^ Furthermore, strong intercorrelations among plasma biomarkers highlight the potential utility of composite ratios, particularly those normalized to Aβ42, in monitoring treatment response.^29^ The high correlation between p‐tau181/Aβ42 and p‐tau217/Aβ42, along with their associations with injury‐ and inflammation‐related ratios, underscores the interplay between amyloid, tau, and glial pathways in AD.^30^ The strong positive correlation between NfL and GFAP further suggests coordinated mitigation of neuroaxonal damage and astrocytic activation during treatment. These findings highlight the coordinated response of multiple pathological processes to lecanemab treatment and support the utility of composite biomarkers, particularly Aβ42‐normalized ratios, for tracking treatment effects in anti‐amyloid therapy.

Regarding safety, concerns over ARIA remain central to clinical adoption. In Clarity AD, ARIA occurred in 21.3% of the overall population (ARIA‐E 12.6%, ARIA‐H 17.3%) and was more frequent among *APOE* ε4 homozygotes. A real‐world study in the United States reported lower ARIA rates of 22% overall (ARIA‐E 15%, ARIA‐H 6.7%), possibly due to smaller brain volumes and different amyloid kinetics in Asian populations.^31^ In our cohort, ARIA incidence was 9.2% (ARIA‐H 8%, ARIA‐E 1.6%). *APOE* ε4 carriers remain at significantly increased risk – consistent with meta‐analyses confirming a two‐fold higher likelihood of ARIA – highlighting the importance of pretreatment genetic profiling for personalized risk–benefit assessment.^4^, ^32^

Compared with pivotal randomized controlled trials, our study extends evidence to a broader disease spectrum, including moderate AD, where ARIA incidence was comparable to early‐stage disease, suggesting acceptable safety in this subgroup.^33^ Our patient group also had higher comorbidity rates and treatment heterogeneity, better reflecting real‐world practice.

From a health policy and cost‐effectiveness perspective, lecanemab remains excluded from China's National Reimbursement Drug List, resulting in significant financial burden.^34^ Notably, some patients who achieved amyloid negativity within 6 months maintained stable cognition even without continued intensive dosing. Given that the FDA has approved a once‐monthly maintenance regimen after 18 months for lecanemab,^35^ and similar early‐de‐escalation protocols are under investigation for donanemab, there is rationale for exploring earlier transition to maintenance dosing in patients achieving rapid amyloid clearance.^36^

Overall, these findings indicate that, in Chinese real‐world settings, lecanemab produces meaningful pathological and biomarker improvements with an acceptable safety profile across different stages of AD. Monitoring treatment response using the plasma biomarker p‐tau217 could support precision medicine approaches that optimize efficacy, safety, and cost‐effectiveness, thereby facilitating sustainable AD management, particularly in resource‐limited settings.xxx

## FUNDING INFORMATION

This study was supported by a grant from the National Natural Science Foundation of China (No. 82171178, 82371186), Guangzhou Municipal Health Science and Technology Project (No. 2025A031003), Key Medical Discipline of Guangzhou (2025–2027), Technologies R&D Program of Guangzhou (No. 2024A04J3549), Guangdong Association for Brain Science Application (No. BSAA2024‐AD‐01004), Dongguan Science and Technology of Social Development Program (No. 20231800936092), and Brain Health Youth Fund ‐ Precision Diagnosis and Treatment Research for Alzheimer's Disease (2025).

Data collection and sharing for the Alzheimer's Disease Neuroimaging Initiative (ADNI) is funded by the National Institute on Aging (National Institutes of Health Grant U19AG024904). The grantee organization is the Northern California Institute for Research and Education. In the past, ADNI has also received funding from the National Institute of Biomedical Imaging and Bioengineering, the Canadian Institutes of Health Research, and private sector contributions through the Foundation for the National Institutes of Health (FNIH) including generous contributions from the following: AbbVie, Alzheimer's Association; Alzheimer's Drug Discovery Foundation; Araclon Biotech; BioClinica, Inc.; Biogen; BristolMyers Squibb Company; CereSpir, Inc.; Cogstate; Eisai Inc.; Elan Pharmaceuticals, Inc.; Eli Lilly and Company; EuroImmun; F. Hoffmann‐La Roche Ltd. and its affiliated company Genentech, Inc.; Fujirebio; GE Healthcare; IXICO Ltd.; Janssen Alzheimer Immunotherapy Research & Development, LLC.; Johnson & Johnson Pharmaceutical Research & Development LLC.; Lumosity; Lundbeck; Merck & Co., Inc.; Meso Scale Diagnostics, LLC.; NeuroRx Research; Neurotrack Technologies; Novartis Pharmaceuticals Corporation; Pfizer Inc.; Piramal Imaging; Servier; Takeda Pharmaceutical Company; and Transition Therapeutics.

## CONFLICT OF INTEREST STATEMENT

The authors declare no conflict of interest.

## CONSENT STATEMENT

The study was approved by the Clinical Research and Application Ethics Committee of the Second Affiliated Hospital of Guangzhou Medical University (No. KY‐EC2024‐049‐01). All participants and study partners provided written consent.

## Supporting information



Supporitng Information

Supporitng Information
