# Supplementary material for: Effectiveness, safety, and biomarker dynamics of lecanemab in Chinese Alzheimer's disease population: a multicenter real‐world study
Source: Alzheimers Dement. 2026 Mar 13;22(3):e71231. doi: 10.1002/alz.71231 (PMC13093726; doi:10.1002/alz.71231)
Supplement: Supplementary file 1 — Supporitng Information [file ALZ-22-e71231-s002.docx]

**Table S1. Demographic characteristics of ADNI and Lecanemab matched cohort**

|  | **PSM-ADNI (N=76)** | **PSM-Lecanemab (N=60)** | **P** |
| --- | --- | --- | --- |
| **Age, Mean (SD)** | 71.2 (7.38) | 70.1 (8.58) | 0.50 |
| **Education, N (%)** |  |  | 0.70 |
| 0-12 years | 42 (55.3%) | 36 (60.0%) |  |
| >12 years | 34 (44.7%) | 24 (40.0%) |  |
| **Gender, N (%)** |  |  | 0.85 |
| Female | 42 (55.3%) | 35 (58.3%) |  |
| Male | 34 (44.7%) | 25 (41.7%) |  |
| **APOE ε4 status, N (%)** |  |  | 0.56 |
| Noncarrier | 30 (39.5%) | 25 (41.7%) |  |
| Carrier |  |  |  |
| Heterozygous | 35 (46.1%) | 29 (48.3%) |  |
| Homozygous | 11 (14.5%) | 6 (10.0%) |  |
| **CDR-GS, N (%)** |  |  | <0.01 |
| 0.5, MCI | 64 (84.2%) | 24 (40.0%) |  |
| 1, Mild AD | 12 (15.8%) | 24 (40.0%) |  |
| 2, Moderate AD | 0 (0%) | 12 (20.0%) |  |

**Table S2. Demographic and clinical characteristics of participants at different time points**

| **Characteristics** | **Baseline (N=261)** | **3 months (N=82)** | **6 months (N=50)** | **P** |
| --- | --- | --- | --- | --- |
| **Age, Mean (SD)** | 69.74 (8.92) | 71.7 (9.1) | 70.9 (8.5) | 0.23 |
| **Education, N (%)** |  |  |  | 0.62 |
| 0-12 years | 66 (25.3) | 55 (67.1) | 30 (60.0) |  |
| >12 years | 195 (74.7) | 26 (31.7) | 20 (40.0) |  |
| **Gender, N (%)** |  |  |  | 0.51 |
| Female | 154 (59.0) | 51 (62.2) | 33 (66.0) |  |
| Male | 107 (41.0) | 30 (36.6) | 17 (34.0) |  |
| **BMI, Mean (SD)** | 22.40 (2.99) | 22.4 (3.1) | 22.0 (2.5) | 0.74 |
| **APOE ε4 status, N (%)** |  |  |  | 0.24 |
| Noncarrier | 105 (40.2) | 33 (40.2) | 15 (30.0) |  |
| Carrier | 125 (47.89) | 45 (54.9) | 36 (72.0) |  |
| Heterozygotes | 105 (40.2) | 40 (48.8) | 29 (58.0) |  |
| Homozygotes | 20 (7.7) | 5 (6.1) | 7 (14.0) |  |
| **Current use of medication, N (%)** |  |  |  |  |
| Acetylcholinesterase inhibitor | 126 (48.3) | 63 (76.2) | 40 (80.0) | <0.01 |
| NMDA receptor antagonist | 74 (28.4) | 37 (45.1) | 21 (42.0) | 0.06 |
| Antiplatelet agents | 30 (11.5) | 18 (22.0) | 5 (10.0) | 0.13 |
| Anticoagulant | 2 (0.7) | 0 (0) | 0 (0) | >0.99 |
| Antihypertensives | 65 (24.9) | 21 (25.6) | 8 (16.0) | 0.07 |
| Antidiabetic agents | 30 (11.5) | 17 (20.7) | 11 (22.0) | 0.31 |
| Antipsychotics | 20 (7.7) | 5 (6.1) | 4 (8.0) | 0.19 |
| None | 97 (37.2) | 9 (11.0) | 2 (4.0) | <0.01 |
| **Comorbidities, N (%)** |  |  |  |  |
| Hypertension | 65 (24.9) | 33 (40.2) | 16 (32.0) | 0.10 |
| Diabetes | 30 (11.5) | 17 (20.7) | 11 (22.0) | 0.36 |
| CVDs* | 27 (10.3) | 12 (14.6) | 5 (10.0) | 0.25 |
| Anxiety | 5 (1.9) | 4 (4.9) | 3 (6.2) | 0.16 |
| Depression | 3 (1.1) | 1 (1.2) | 1 (2.0) | 0.81 |
| Atrial fibrillation | 2 (0.7) | 1 (1.2) | 0 (0) | 0.71 |
| None | 137 (52.5) | 6 (7.3) | 1 (2.0) | <0.01 |
| **MMSE, Mean (SD)** | 18.22 (6.10) | 18.1 (5.9) | 16.9 (6.1) | 0.22 |
| **CDR-GS, N (%)** |  |  |  | 0.86 |
| 0.5, MCI | 107 (41.0) | 30 (36.6) | 17 (34.0) |  |
| 1, Mild AD | 108 (41.4) | 36 (43.9) | 21 (42.0) |  |
| 2, Moderate AD | 46 (17.6) | 16 (19.5) | 11 (22.0) |  |
| **Microhemorrhage, N (%)** | 37 (14.2) | 30 (36.6) | 22 (44.0) | <0.01 |
| **Superficial siderosis, N (%)** | 4 (1.5) | 3 (3.6) | 3 (6.0) | 0.10 |

**Table S3. Outcomes of participants undergoing Aβ** **PET reassessment**

| **Outcomes** | **Before treatment**  **(N=29)** | **After 6 months of treatment**  **(N=29)** | **P** |
| --- | --- | --- | --- |
| **Clinical Outcome, Mean (SD)** |  |  |  |
| CDR-SB | 4.82 (3.24) | 5.16 (3.04) | 0.39 |
| MMSE | 16.52 (6.16) | 18.21 (6.92) | 0.03 |
| ADCS-MCI-ADL | 32.12 (10.98) | 31.67 (11.49) | 0.74 |
| ADAS-Cog14 | 34.29 (14.34) | 39.73 (17.13) | 0.02 |
| HAMD | 6.75 (4.51) | 5.17 (5.21) | 0.11 |
| NPI | 1.08 (1.84) | 2.17 (3.10) | 0.11 |
| **Plasma Biomarkers, Mean (SD)** |  |  |  |
| Aβ42 (pg/ml) | 3.787 (1.84) | 5.46 (2.21) | <0.01 |
| Aβ40 (pg/ml) | 80.67 (46.16) | 130.55 (53.88) | <0.01 |
| Aβ42/40 | 0.047 (0.013) | 0.043 (0.012) | 0.58 |
| P-Tau217 (pg/ml) | 1.20 (0.76) | 1.028 (0.61) | 0.68 |
| P-Tau181 (pg/ml) | 3.06 (1.79) | 2.848 (1.53) | 0.71 |
| GFAP (pg/ml) | 246.82 (121.90) | 199.98 (134.05) | 0.34 |
| NfL (pg/ml) | 22.53 (9.53) | 20.321 (10.95) | 0.71 |
| P-Tau181/Aβ42 | 0.88 (0.52) | 0.52 (0.37) | <0.01 |
| P-Tau217/Aβ42 | 0.30 (0.17) | 0.20 (0.13) | 0.02 |
| GFAP/Aβ42 | 66.42 (44.82) | 44.30 (42.54) | 0.01 |
| NfL/Aβ42 | 4.90 (1.90) | 4.27 (4.28) | 0.2 |
| **PET Burden, Mean (SD)** |  |  |  |
| Summary Aβ (Centiloid) | 49.79 (25.76) | 27.75 (22.12) | 0.02 |
| Cortex Tau, N=16 (SUVR) | 1.35 (0.30) | 1.35 (0.32) | 0.85 |
